# Advancing prenatal diagnosis: Echocardiographic detection of Scimitar syndrome in China – A case series

**DOI:** 10.1515/med-2024-0989

**Published:** 2024-07-05

**Authors:** Yan Wang, Bin Geng, Peizhi Yang, Wenxiu Li

**Affiliations:** The Department of Ultrasound, Children’s Hospital of Shanxi Province (Maternal and Child Health Hospital of Shanxi Province), Taiyuan, China; Pediatric Cardiovascular Center, Beijing Anzhen Hospital, Capital Medical University, 2 Anzhen Road, Chaoyang District, Beijing, 100029, China

**Keywords:** echocardiography, fetus, Scimitar syndrome, pulmonary hypoplasia, cardiac malposition

## Abstract

**Objective:**

To investigate the clinical value of echocardiographic detection in the prenatal early diagnosis of Scimitar syndrome (SS) in fetuses, and to develop better and more accurate management strategies for improved prognosis.

**Methods:**

A retrospective analysis was conducted on medical records and fetal echocardiographic findings of all cases diagnosed as SS between April 1, 2016 and June 1, 2021. To summarize its echocardiographic features and distinguishing points, comprehensive clinical data and prognostic information were gathered.

**Results:**

Six patients were diagnosed with SS during the study period. Major associated abnormalities included atrial septal defect (*n* = 3), right inferior pulmonary vein anomalies (*n* = 2), ventricular septal defect (*n* = 1), and right aortic arch (*n* = 1). Post-surgery, all patients exhibited unobstructed pulmonary vein flow and absence of pulmonary hypertension. The average follow-up duration was 24 months, during which five infants underwent surgical intervention for SS.

**Conclusion:**

Comprehensive prenatal screening, particularly combined coronal and sagittal views of the fetal thorax, enables accurate diagnosis of right SS. This approach not only aids in timely intervention but also provides crucial prognostic insights for the child’s future well-being.

## 1 Introduction

Scimitar syndrome (SS) is a rare congenital anomaly, with an estimated incidence of 1–3 in 100,000 live births, including partial or complete abnormalities of right or left pulmonary veins (LPV) draining to the inferior vena cava (IVC) above or below the diaphragm, hypoplastic right pulmonary artery and lung, aortopulmonary collateral(s) to the right lung, and bronchial anomalies [1,2]. It was initially described in 1836 based on the autopsy of an infant with dextrocardia and right lung hypoplasia. Subsequently, it was reported for the second time in 1949 in an asymptomatic living patient through cardiac catheterization [3]. The first reported surgical intervention occurred in 1950 in a patient with recurrent pneumonia, with the initial corrective surgery performed in 1956 [3]. However, it was not until 1960 that the syndrome was formally named [4], and its association with radiological findings was established. The anomalous vein was initially described using the term “scimitar” as its radiographic appearance resembles the short-curved scimitar sword. Specifically, the anomalous return of pulmonary veins into the right heart system (right atrium [RA] or RA through the IVC) leads to an increase in the volume of the right heart system, which is manifested by the enlargement of the RA and right ventricle and the widening of the pulmonary artery, i.e., the increase in the blood volume of the right heart system. Because of the influence of the fetal position in the uterus and the pregnant woman’s own obesity factors, even if the changes of the fetal heart and hemodynamics are observed, it is difficult to determine the number of anomalous pulmonary venous drainage, the drainage route, and the place where the final drainage will pass. The ultrasound presentation is more complex. Especially, it is very difficult to fully display the pulmonary venous drainage route, and it is difficult to display it on the conventional section. However, there are few reports of prenatal diagnosis in China.

SS symptoms encompass cyanosis, respiratory distress, tachypnea, recurrent pneumonia, and heart failure. Additionally, SS presents in two distinct forms: infantile, manifesting early in life, often symptomatic and requiring intervention, and adult, typically asymptomatic [5]. As reported by Gao et al., more severe cases of SS occur early in life, often due to abnormal arterial collaterals supplying the RA, leading to increased pulmonary blood flow and respiratory distress [6]. Up to now, most cases of infants described in the medical literature have been diagnosed in the second half of pregnancy or in the postpartum period. The more severe cases of SS present early in life, usually with dyspnea due to increased pulmonary blood flow secondary to the abnormal collateral artery supplying systemic arterial blood to the RA. Postnatal diagnosis helps the physician confirm the diagnosis based on imaging such as computed tomography or angiography [7–9].

In summary, many studies have been proposed to diagnose SS by prenatal echocardiography, mainly in the middle of pregnancy. However, reaching a diagnosis prenatally remains a challenge [10,11]. Here, we present six cases of early prenatal diagnosis of SS at 26–32 weeks of gestation, as well as outline the shared characteristic sonographic findings in the fetal period by comparing the prenatal echocardiographic characteristics. The objective is to enhance clinical understanding of SS among healthcare professionals and supplement research on the treatment of SS in Chinese centers.

## 2 Materials and methods

Computerized records of all fetuses that had undergone ultrasound examinations between April 2016 and June 2021 at the Pediatric Cardiovascular Center, Beijing Anzhen Hospital, China, were analyzed for cases of SS. Unilateral pulmonary dysplasia caused by other extracardiac malformations was excluded. All cases were retrospectively reviewed for intrauterine findings, course, and outcome. A prenatal diagnosis of SS was made in the presence of hypoplasia of the right lung resulting in ipsilateral mediastinal shifting and abnormal pulmonary venous drainage to the IVC. All cases underwent a complete fetal anatomic survey that included fetal echocardiography and Doppler sonography in a standardized fashion. Fetal echocardiography was carried out by a segmental approach using standardized anatomical planes incorporating pulsed-wave and color Doppler imaging. All fetal echocardiographic examinations were performed on Philips IE33, Philips EPIQ 7C (Philips Royal Electronics Corporation, Holland), or GE Voluson E8 (GE Healthcare, Kretztechnik, Zipf, Austria) Color Doppler ultrasound systems using 4–8 MHz transabdominal transducer. Examinations were done by the 2013 guidelines of the American Institute of Ultrasound in Medicine [12]. Two radiologists were blinded to any patient’s clinical data to mitigate potential cognitive biases. Using medical record data can mitigate selection bias and provide detailed prognostic information for individual cases. Diagnoses of right-sided SS were confirmed by postnatal echocardiography and/or surgery in all cases.

The following prenatal parameters were collected: maternal age and gestational age at referral, the heart position, cardiac axis, cardiothoracic ratio, echo, and area of the bilateral lung. Postnatal medical records such as surgical, ultrasonic, and clinical findings were reviewed as well. Simultaneous two-dimensional monitoring of esophageal echocardiography was performed intraoperatively in all patients to evaluate in real time whether the postoperative pulmonary veins were draining into the left atrium, whether the blood flow was patent, and whether the other malformations were corrected satisfactorily at the same time. Three to four different bedside echocardiograms were performed during the hospital stay to clarify whether the postoperative cardiac function and hemodynamics were normal, whether pulmonary veins were stenotic, whether resistance type pulmonary hypertension was present, and whether pleural and abdominal effusions were present. Patients were followed up by echocardiography, ECG, and chest X-ray at the outpatient clinic on the first, third, sixth, twelfth, and every other year after discharge. Echocardiography was performed mainly to assess the position of the pulmonary venous drainage and the patency of the blood flow and the presence or absence of pulmonary venous stenosis by measuring the reflux velocity and pressure difference of the pulmonary veins.

The coronal and sagittal views of the fetal thorax were included in this study. Pulsed, color, and power Doppler interrogation were also conducted to confirm the location of pulmonary venous drainage, and the systemic arterial blood supply arising from the aorta. All fetal echocardiography examinations were performed and reviewed by two fetal/pediatric cardiologists.


**Ethical approval:** The study was approved by the Ethics Committee of Beijing Anzhen Hospital. The IRB number was 2020038X.
**Informed consent:** Informed consent was obtained from all participants before the study.

## 3 Results

### 3.1 General characteristics of the mother and the fetuses

During the period, a total of 1,420 fetuses with congenital heart disease (CHD) were examined in our center, and six cases were referred to our center for fetal echocardiography at 26–32 weeks gestation because of suspected CHDs. The reasons for their referral were all cardiac malformations. The age of pregnant women ranged from 26 to 35 years. All couples were healthy and nonconsanguineous, and all pregnant women had unremarkable family histories. None of the cases had extra-cardiac findings.

The right inferior pulmonary vein drained directly into the IVC below the diaphragm in cases 1 and 6, while the others involved the complete right pulmonary veins. In case 2, pulmonary vein reflux entered the superior aspect of the right atrial orifice of the IVC. All three cases presented with concomitant atrial septal defects (ASDs) postnatally. Case 4 was diagnosed postnatally with echocardiography and cardiac computed tomography angiography, revealing the simultaneous occurrence of the left lower pulmonary artery originating from the distal right pulmonary artery. In case 5, echocardiography revealed significant tricuspid regurgitation accompanied by severe pulmonary hypertension. The cardiac axis was 0° in case 2 and other fetuses had normal cardiac axis. On the cross-sectional four-chamber view, the area of the dysplastic lung was about 1/2 to 1/3 of the contralateral normal lung area. Table 1 summarizes the clinical observations.

**Table 1:** Abnormalities and outcomes of six cases with right-sided scimitar syndrome

Case	MA (years)	GA (weeks)	The heart position	Cardiac axis	Affected lung	Pulmonary veins drainage	Prenatal ultrasound	Associated heart anomalies	Outcome/surgery
1	26	26	Extraposition	Left axis 35°	R	RIPV-IVC	SS	ASD	Full-term delivery, surgery, alive
2	29	26	Mesocardia	0°	R	RPV-IVC	SS, VSD, RAA	VSD, RAA	Full-term delivery, surgery, alive
3	28	32	Dextroposition	Left axis 40°	R	RPV-IVC	SS	—	Full-term delivery, surgery, alive
4	35	28	Dextroposition	Left axis 35°	R	RPV-IVC	SS, PLSVC	PLSVC, ASD, partial pulmonary sling	Full-term delivery, surgery, alive
5	33	26	Dextroposition	Left axis 30°	R	RPV-IVC	SS, PLSVC	ASD, PLSVC	Full-term delivery, surgery, alive
6	31	30	Dextroposition	Left axis 40°	R	RIPV-IVC	SS, PE	—	Full-term delivery, not yet operated, alive

ASD = atrial septal defect; GA = gestational age; IVC = inferior vena cava; MA = maternal age; PE = pericardial effusion; PLSVC = persistent left superior vena cava, R = right; RAA = right aortic arch; RIPV = right inferior pulmonary vein; RPV = right pulmonary vein; SS = scimitar syndrome; VSD = ventricular septal defect.

### 3.2 Prenatal ultrasound findings

The common sonographic findings include: (1) shifting of the heart toward the right dysplastic lung, (2) hypoplastic right pulmonary artery branch and small lung area on the right dysplastic side, (3) normal and consistent appearance of lung tissue on echo, and (4) the coronal and sagittal view of fetal thorax being clearer for the site and course of pulmonary venous drainage. Three of the six fetuses presented with ASD, two fetuses with right inferior pulmonary vein, one fetus with ventricular septal defect, and one fetus with right aortic arch (Table 1).

**Figure 1:**
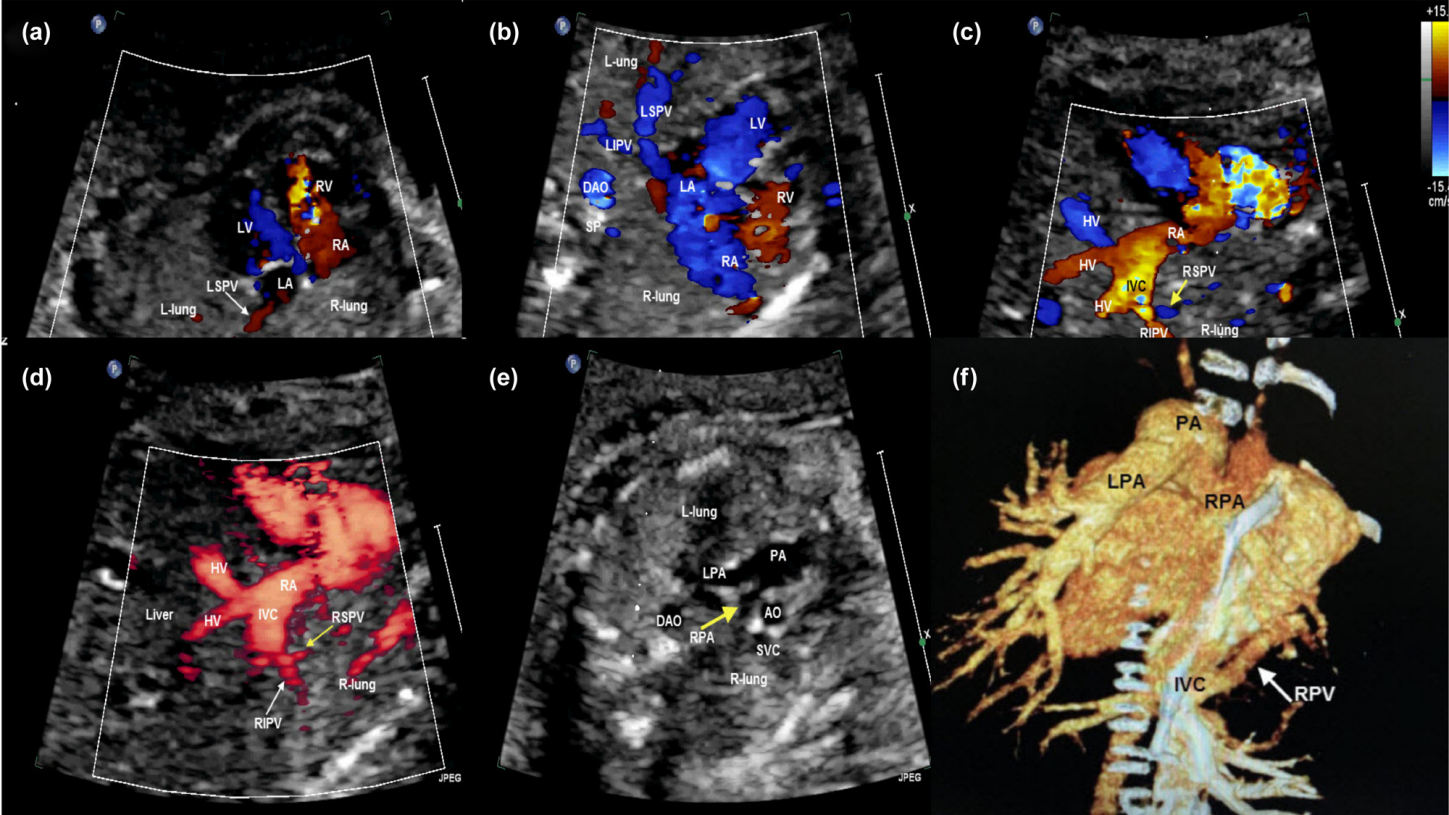
Fetal echocardiogram of case 2 at the time of diagnosis. (a) The four-chamber view demonstrated the heart was myocardial and the heart axis shifted to the right, the echo of bilateral lung tissue was consistent and normal, and the right lung was dysplastic. (b) Color Doppler showing the LPV flowed back into the LA, but no normal right pulmonary vein flowed back into the LA. (c) Color Doppler demonstrating the right pulmonary veins (yellow arrow) drained into the IVC on sagittal view. (d) Power Doppler demonstrating the right pulmonary veins (white and yellow arrows) drained into the IVC on sagittal view. (e) The bifurcation of the PA view demonstrating that the origin of the pulmonary branch was normal and the inner diameter of the right PA was narrow (yellow arrow). (f) Cardiac CT demonstrating the LPV (white arrow) flowed back into the LA, and the right PA was narrow. AO, aorta; DAO, descending aorta; HV, hepatic vein; IVC, inferior vena cava; LA, left atrium; LIPV, left inferior pulmonary vein; L-lung, left-lung; LPA, left pulmonary artery; LSPV, left superior pulmonary vein; LV, left ventricle; PA, pulmonary artery; RA, right atrium; RIPV, right inferior pulmonary vein; R-lung, right-lung; RPA, right pulmonary artery; RSPV, right superior pulmonary vein; RV, right ventricle; SP, spine.

**Figure 2:**
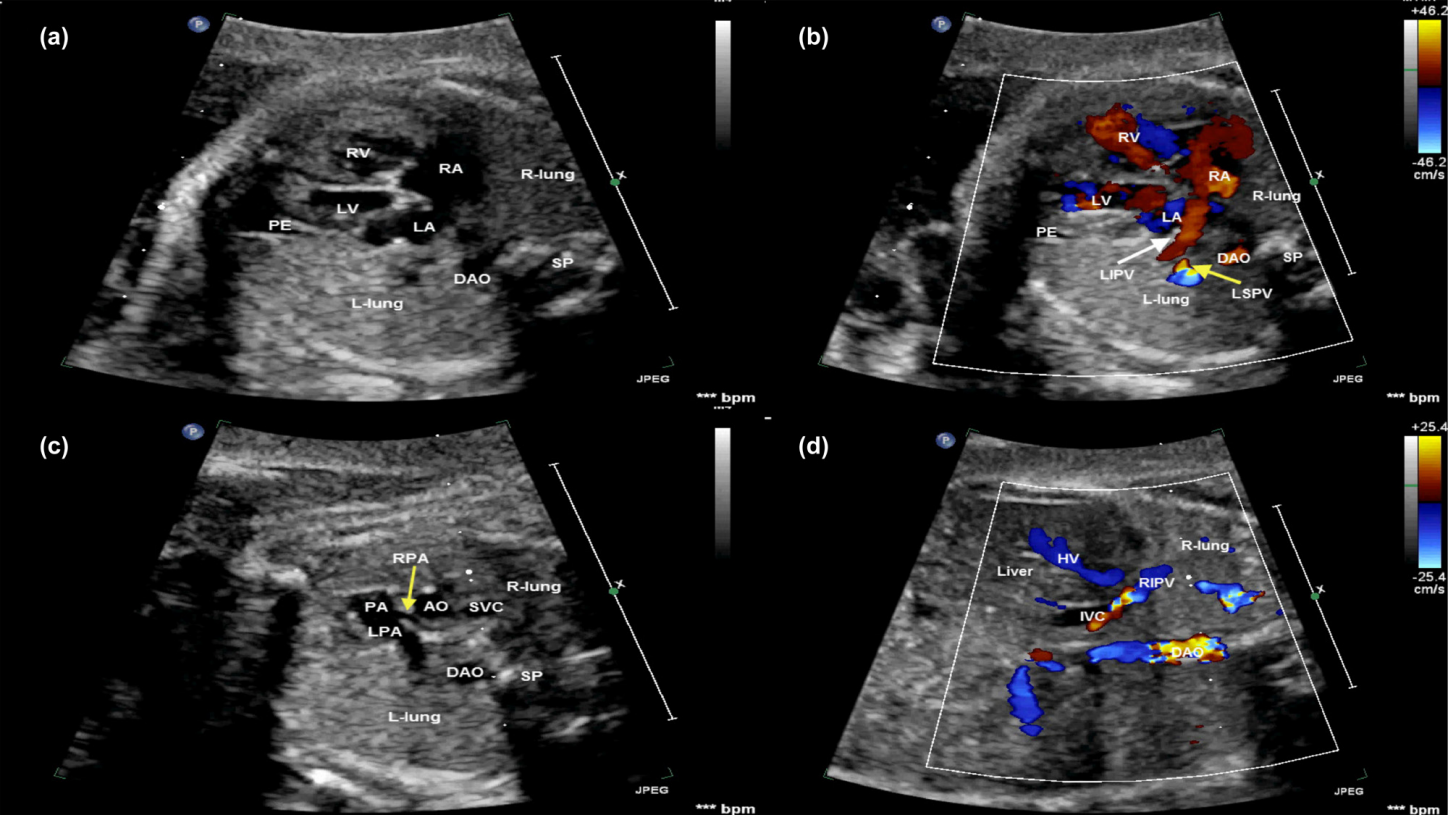
Fetal echocardiogram of case 6 at the time of diagnosis. (a) The four-chamber view demonstrated the heart shifted to the right, the echo of bilateral lung tissue was consistent and normal, and the right lung was dysplastic. A small PE was seen at the apex of the LV. (b) Color Doppler showing the LPV (white and yellow arrows) flowed back into the LA and no normal right pulmonary vein flowed back into the LA. (c) The bifurcation of the PA view demonstrating that the origin of the pulmonary branch was normal and the inner diameter of the RPA (yellow arrow) was narrow than the left PA. (d) Color Doppler showing the RIPV passed through the diaphragm and drained into the IVC on a coronal view. AO, aorta; DAO, descending aorta; HV, hepatic vein; IVC, inferior vena cava; LA, left atrium; LIPV, left inferior pulmonary vein; L-lung, left-lung; LPA, left pulmonary artery; LV, left ventricle; PA, pulmonary artery; RA, right atrium; RIPV, right inferior pulmonary vein; R-lung, right-lung; RPA, right pulmonary artery; RSPV, right superior pulmonary vein; RV, right ventricle; SP, spine.

All sonographic findings are characterized by color and power Doppler imaging, demonstrating the affected right pulmonary vein draining into the IVC below the diaphragm or the junction of the IVC and the RA on the coronal and sagittal views of the thorax in all fetuses (Figures 1 and 2). A solitary small abdominal aortopulmonary collateral was found by the prenatal echocardiography only in case 6.

### 3.3 Postnatal course and follow-up

The fetuses were spontaneously delivered at full terms. An ostium secundum ASD was diagnosed after birth in cases 1 and 4. Additionally, in case 4, the prenatal ultrasound missed the diagnosis of the left inferior pulmonary artery arising from the distal part of the right pulmonary artery (partial pulmonary sling). Other cardiac anomalies were confirmed by the postnatal echocardiography examinations, cardiac-enhanced CT examinations, and surgery for cardiac anomalies. Postnatal examinations displayed abnormal arterial supply arising from the aorta in hypoplasia lung only in case 6, who is not operated on yet, but the investigator has been on regular follow-up visits for this patient. The main clinical symptoms were shortness of breath in cases 1 and 5 and recurrent chest infections in case 2.

Right pulmonary vein reconstruction and surgeries for other cardiac anomalies were performed in five cases approximately 4–9 months after birth, and all warranted embolization of one or more abnormal arteries that supplied the affected hypoplastic right lung. They were discharged from the hospital without complications 10–20 days after surgery. The diagnosis was confirmed by postnatal echocardiography in case 6, and the patient was asymptomatic. The patient has been under close periodic observation. At the time of data collection, all children were alive and in good health, at a 3–24-months follow-up.

## 4 Discussion

The most important indicator for prenatal diagnosis of SS was the abnormal position of the thoracic heart with mediastinal shift on the right side. In these cases, definitive abnormal pulmonary venous drainage was necessary to establish the diagnosis and differentiate dextrocardia from other causes. It was characterized by partial or complete anomalous right or left pulmonary venous drainage to the IVC, the junction of the IVC and the RA or the lower part of the RA, hypoplastic right pulmonary artery and lung, aortopulmonary collateral(s) to the right lung, and bronchial anomalies [1,8]. In the present study, early diagnosis of the above-mentioned abnormalities was performed by prenatal ultrasound, and early diagnosis and clinical analysis were performed postpartum.

The prevalence of SS ranged from 0.001 to 0.003%, rendering it an exceedingly rare cardiovascular anomaly. It represented a subtype of partial anomalous pulmonary venous return. Its features include partial or complete anomalous drainage of the right or, rarely, LPV into the IVC, at the confluence of the IVC and RA or at a lower position within the RA. In most instances, blood flow from the entire right lung was directed outward via the “scimitar” vein [1,2,13]. Grisaru et al. [14] reported a prenatally diagnosed case of SS, highlighting the importance of considering SS in cases of fetal cardiac rightward displacement and underdeveloped right pulmonary artery. Diagnosis often occurs late in pregnancy due to limited literature and ultrasound experience. However, recent studies by Khatib et al. described early diagnosis at 14–16 weeks of pregnancy, underscoring the significance of cardiac rightward displacement and underdeveloped right pulmonary artery as indicators. Confirmation of SS diagnosis may involve identifying the “scimitar” vein behind the IVC and normal lung tissue echoes. It is recommended to conduct slow, continuous scanning from the chest to the upper abdomen to visualize the descending “scimitar” vein or single pulmonary vein. Previous research findings align with ours. Paladini et al. [15] summarized four prenatal series comprising 13 cases, of which ten (76.9%) survived. Among these, six cases did not require surgery, and associated anomalies were rare, representing the milder end of the spectrum. In our study, one out of six cases did not undergo surgery, and postoperative recovery was satisfactory. This correlates with our emphasis on early diagnosis to facilitate timely intervention. However, an Indian study revealed that 3 out of 4 cases of SS exhibited associated cardiac and extracardiac defects, particularly incomplete right lung development. The surgical mortality rate ranged from 4.8 to 5.9%, possibly attributed to the higher prevalence of comorbidities. In our study, the majority of patients were asymptomatic during the late clinical examination, possibly because they exhibited only cardiac abnormalities while other prenatal examinations were normal, and the follow-up duration was short, lasting only 2 years. Clinical decisions regarding the need for follow-up surgery were based on postnatal fetal monitoring, evaluation, and follow-up assessments. Clinical symptoms typically manifested approximately 4–9 months after birth, leading to corrective surgery in five cases involving right pulmonary vein reconstruction and other cardiac anomalies. These patients underwent surgery to correct associated deformities such as ASD, ventricular septal defect, and partial pulmonary slip. They experienced smooth recoveries and were discharged from the hospital without complications within 10–20 days post-surgery. Subsequent follow-ups at 3–24 months revealed normal development with no signs of pulmonary arterial hypertension or pulmonary vein stenosis. Long-term lifelong follow-up remains necessary after surgery, with additional surgical intervention being considered if required.

Our study, after summarizing the ultrasonic characteristics of six cases of SS, found that during routine cardiac scanning, attention should be paid to the position of the heart. When the heart was displaced to the right, examiners need to probe bilaterally and from multiple angles to determine if the pulmonary venous return is normal. The confluence of the IVC and the right atrial orifice was a common site where the “scimitar” vein, formed by the right pulmonary veins, or two separate right pulmonary veins draining abnormally through the diaphragm, was found. Therefore, in addition to routine apical four-chamber and transverse four-chamber scanning, it was essential to focus on the coronal and sagittal sections of the IVC, which clearly display the position of anomalous pulmonary venous drainage, crucial for diagnosing SS. Additionally, SS often involved collateral circulation from the abdominal aorta, descending aorta, or celiac artery supplying the right lung lobe. Children with these systemic-to-pulmonary arterial collaterals may present with respiratory symptoms postnatally and may require embolization of these collaterals. As these systemic-to-pulmonary arterial collaterals were clearly visible during fetal life, prenatal diagnosis was also essential After analyzing and summarizing the prenatal echocardiographic characteristics of all fetuses in this study, consideration of the diagnosis of SS on ultrasound was warranted by every obstetrician and gynecologist when heart malposition was detected during prenatal ultrasound examinations, provided there was no suspicion for bronchopulmonary sequestration and congenital diaphragmatic hernia, as reported previously [7,14,16]. Based on this characteristic, a detailed scan of the pulmonary vein, main pulmonary artery, and branches of the pulmonary artery was performed to determine the presence or absence of pulmonary vein drainage. For fetuses suspected of having SS, spectral, color, and power Doppler imaging were utilized to demonstrate anomalous pulmonary venous drainage and the drainage location on the coronal and sagittal views of the fetal thorax. A chest radiograph could reveal the complete course of the right pulmonary vein in SS, making the coronal and sagittal views of the thorax crucial for diagnosis [17]. The color Doppler range was reduced, and the gain was increased in the examination of the pulmonary vein in fetuses with small gestational age. Visualization of a confluence behind the RA and a vertical vein provided the most consistent echocardiogram clue [18].

Additionally, pulmonary dysplasia predominantly affected the right lung tissue, consistent with previous reports [1,2]. It was crucial to emphasize that pulmonary hypoplasia led to abnormal pulmonary vein development on the ipsilateral side [19]. However, underdevelopment of the main pulmonary artery and its branches did not necessarily coexist with pulmonary dysplasia, as seen in cases of tetralogy of Fallot, Williams syndrome, and pulmonary atresia [1,11]. Treatment for SS included drug therapy, interventional therapy (pulmonary embolism), and surgical treatment [4,15,20,21]. Based on the onset of clinical symptoms, SS could be classified into infantile and childhood/adult forms. The infantile form typically presented within the first 2 months of life with symptoms such as tachypnea, recurrent pneumonia, failure to thrive, and signs of heart failure [1]. Infantile form represented a severe manifestation of the disease with a poorer prognosis, primarily linked to cardiac lesions and pulmonary hypertension. Previous studies on prenatal diagnosis of SS had yielded variable results.

However, our study still had limitations as it was a case series, with variations in therapeutic techniques used, follow-up duration, and population demographics. Additionally, the population enrolled in the current study was not sufficiently large, highlighting the need for further research involving personalized management with a larger population of SS on a prospective basis. Future studies should aim to collect additional cases for further detailed analysis. Given the small sample size, which restricts the generalizability of the study findings, we will explore extending the study duration or collaborating with other centers to increase the sample size and improve the statistical power of the research.

In conclusion, our study underscores the critical importance of early surgical intervention in improving outcomes for patients/neonates diagnosed with SS, particularly considering the associated cardiovascular malformations. Despite the inherent limitations stemming from the small sample size, the findings emphasize the imperative for accurate prenatal diagnosis during routine obstetric ultrasound examinations. This proactive approach enables timely interventions, which are pivotal for optimizing prognosis in affected individuals.
